# Mutation Screening of Six Exons of *ABCA4* in Iranian Stargardt Disease Patients

**DOI:** 10.18502/jovr.v17i1.10170

**Published:** 2022-01-21

**Authors:** Ensieh Darbari, Hamid Ahmadieh, Narsis Daftarian, Mozhgan Rezaei Kanavi, Fatemeh Suri, Hamideh Sabbaghi, Elahe Elahi

**Affiliations:** ^1^School of Biology, College of Science, University of Tehran, Tehran, Iran; ^2^Opthalmic Research Center, Research Institute for Ophthalmology and Vision Science, Shahid University of Medical Sciences, Tehran, Iran; ^3^Ocular Tissue Engineering Research Center, Research Institute for Ophthalmology and Vision Science, Shahid Beheshti University of Medical Sciences, Tehran, Iran; ^4^Ophthalmic Epidemiology Research Center, Research Institute for ophthalmology and Vision Science, Shahid Beheshti University of Medical Sciences, Tehran, Iran; ^5^Department of Optometry, School of Rehabilitation, Shahid Beheshti University of Medical Sciences, Tehran, Iran

**Keywords:** ABCA4, Mutation Screening, Retinal Dystrophy, Stargardt Disease, STGD1

## Abstract

**Purpose:**

Stargardt disease type 1 (STGD1) is a recessively inherited retinal disorder that can cause severe visual impairment. *ABCA4* mutations are the usual cause of STGD1. *ABCA4* codes a transporter protein exclusively expressed in retinal photoreceptor cells. The genecontains 50 exons. Mutations are most frequent in exons 3, 6, 12, and 13, and exons 10 and 42 each contain two common variations. We aimed to screen these exons for mutations in Iranian STGD1 patients.

**Methods:**

Eighteen STGD1 patients were recruited for genetic analysis. Diagnosis by retina specialists was based on standard criteria, including accumulation of lipofuscin. The six *ABCA4* exons were PCR amplified and sequenced by the Sanger method.

**Results:**

One or more *ABCA4*-mutated alleles were identified in 5 of the 18 patients (27.8%). Five different mutations including two splice site (c.1356+1G > A and c.5836-2A > G) and three missense mutations (p.Gly1961Glu, p.Gly1961Arg, and p.Gly550Arg) were found. The p.Gly1961Glu mutation was the only mutation observed in two patients.

**Conclusion:**

As *ABCA4 *mutations in exons 6, 12, 10, and 42 were identified in approximately 25% of the patients studied, these may be appropriate exons for screening projects. As in other populations, STDG1 causative *ABCA4 *mutations are heterogeneous among Iranian patients, and p.Gly1961Glu may be relatively frequent.

##  INTRODUCTION 

Stargardt disease type 1 (STGD1: OMIM No. 248200) is a relatively common form of macularimpairment of color vision, and degeneration of retinal pigment epithelium (RPE) cells.^[2,3]^ Accumulation of orange–yellow flecks in the macula is often observed during ophthalmoscopic examination. *ATP-binding cassette sub-family A member 4* (*ABCA4*, OMIM: 601691; also known as ABCR) is the most important Stargardt disease-causing gene.^[4,5,6,7,8,9,10]^ This gene is positioned on chromosome 1p21-p22, contains 50 exons, and encodes a 2273 amino acid protein that belongs to ABC transporter protein family. ABC transporter proteins have four essential domains, including two transmembrane domains (TMDs) and two cytoplasmic nucleotide-binding domains (NBDs). TMDs are responsible for translocation of substrates, and NBDs bind to ATP and hydrolyze ATP to ADP to produce energy for the translocation. *ABCA4* expression is specific to the retina and its protein product is responsible for the transport of vitamin A derivatives in the outer segment disc membranes of photoreceptors.^[11,12]^ Mutations in the gene result in accumulation of toxic bisretinoid adducts in RPE cells, eventually leading to RPE cell death and macular degeneration.^[13]^

In addition to STGD1, mutations in *ABCA4 *can cause several other types of retinal degenerative diseases.^[14,15,16]^ This suggests a complex genotype/phenotype relationship between mutations in the gene and the consequent phenotypes. Additionally, the number of sequence variations and disease-associated variations in *ABCA4 *is astounding. Variability in frequencies of the variations in different populations is also notable. The Human Genome Mutation Database (HGMD, http://www.hgmd.cf.ac.uk) reports 1467 mutations in the *ABCA4* gene as cause of various retinal degenerative diseases; 629 of the mutations are associated with STGD1. The Genome Aggregation Database (gnomAD v2.1.1; https://gnomad.broadinstitute.org) reports 3979 sequence variations for *ABCA4*, including exonic, intronic, UTR, splicing, and INDEL variations; the frequency of 3930 of these is < 0.01. The Iranome database (http://www.iranome.ir) which comprises exome sequence data on 800 healthy Iranians reports 389 *ABCA4* sequence variations, and the frequency of 301 of these is < 0.01. Clearly, common sequence variations are unlikely to contribute to disease status, but rare variations may have deleterious effects.

Here, we report the results of mutation screening of six exons of the *ABCA4 *gene in 18 unrelated Iranian Stargardt disease patients. To the best of our knowledge, mutation screening of this gene in Iranians has not been previously reported. Among the 50 exons of *ABCA4*, exons that were more likely to contain mutations were screened. Based on the HGMD database, more mutations have been reported in exons 3, 6, 12, and 13 than in other exons. These exons of *ABCA4 *have, respectively, 33, 33, 39, and 45 reported mutations. In addition to these, exons 10 and 42 were also screened. A common nucleotide sequence variation that causes p.His432Arg is positioned in exon 10. Although now considered a polymorphism, this variation was earlier thought to contribute to disease status. Exon 42 was screened because the most frequent disease-associated variation in various populations is positioned within this exon. This variation causes p.Gly1961Glu.

**Figure 1:**
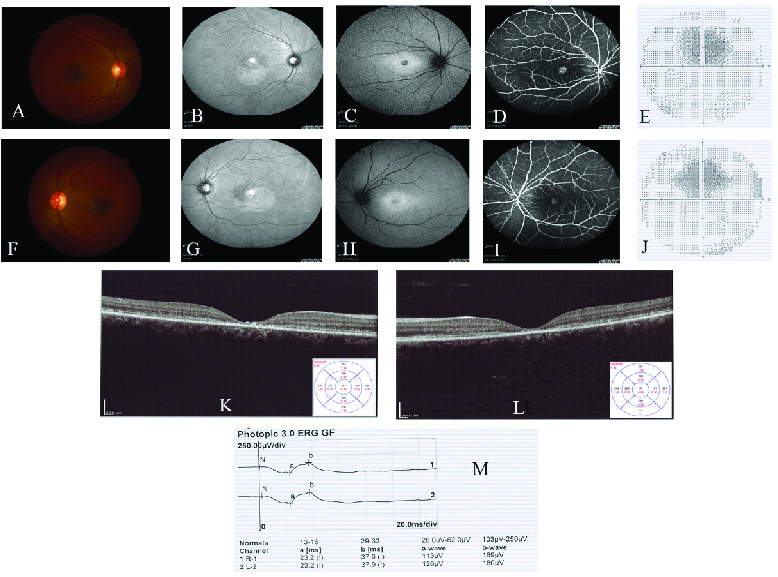
Fundus photographs (color, infrared, and autofluorescence), fluorescein angiography (FA), visual field (VF), optical coherence tomography (OCT), and electroretinography (ERG) findings in a representative patient (STG-5). (A–D) and (F–I) represent color (A & F), infrared (B & G), autofluorescence (C & H), and fluorescein angiographic (D & I) fundus photographs of the patient's right and left eyes, respectively, and demonstrate the lipofuscin accumulation in the macula. VF defects are evident in the right (E) and left eyes (J). Reduced central macular thicknesses were illustrated in the OCT images of the right (K) and left (L) eyes. Note the abnormal photopic ERG graphs and values for both eyes in (M).

**Figure 2:**
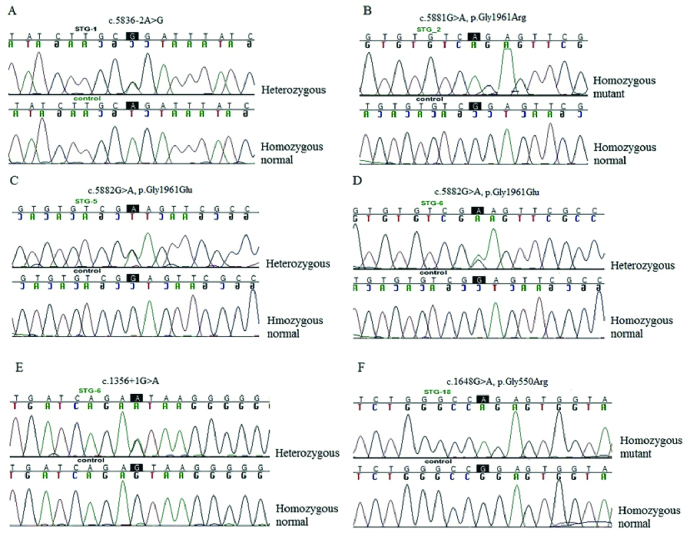
Sequence chromatograms of five mutations observed among the Stargardt disease-affected patients studied. (A) c.5836-2A > G in patient STG-1; (B) c.5881G > A mutation in patient STG-2; (C) c.5882G > A mutation in patient STG-5 and STG-6; (D) mutation c.1356+1G > A in patient STG-6; and (E) mutation c.1648G > A in patient STG-18.

##  METHODS

This research was performed in accordance with the Declaration of Helsinki, with informed consent of participants or responsible guardians, and with the approval of the Ethics Board of the University of Tehran. Eighteen unrelated Stargardt patients were sequentially recruited from the Retina Clinic of Labbafinejad Medical Center affiliated to Shahid Beheshti University of Medical Sciences, Tehran, Iran. Ophthalmic examinations included visual acuity assessment, fundus photographs (color, infrared, and autofluorescence) and fluorescein angiography (FA) for accumulation of lipofuscin in the retina, measurement of macular thickness by optical coherence tomography (OCT), visual field (VF) testing, and electroretinography (ERG). A combination of clinical presentations and progression, decreased visual acuity, fundus photographs, FA, VF, OCT, and ERG results were considered for the diagnosis of Stargardt disease.^[17]^

For genetic analysis, genomic DNA was isolated from the white blood cells of the peripheral blood of the patients. Exons 3, 6, 10, 12, 13, 42, and flanking intronic regions of *ABCA4* were amplified by the polymerase chain reaction (PCR). Primer sequences are available upon request. The amplified PCR products were sequenced using the Sanger sequencing protocol. Sequences were analyzed using the Sequencher Software (Gene Codes Corporation, Ann Arbor, MI). Variations were assessed by comparison with *ABCA4 *reference sequence available at NCBI (NC_000001.10, NM_000350.3, NP_000341.2).

##  RESULTS

The average age of patients at the disease onset was 17 years (range 6–41 years). Of the 18 included patients, 11 (61%) were male. The average age at the examination was 26 years (range 8–45 years). All investigated patients demonstrated decreased best-corrected visual acuity and evidence of lipofuscin accumulation in the macula, decreased central macular thickness, constricted visual fields, and variable degrees of ERG abnormalities [Figure 1].

Of the 18 patients screened, three (STG-2, STG-6, and STG-18) had two definitive disease-causing mutated *ABCA4 *alleles [Table 1]. Of these, two patients had homozygous mutations (p.Gly1961Arg and p.Gly550Arg), consistent with them having been born to consanguineous parents. The third patient had compound heterozygous mutations; one was the very common p.Gly1961Glu-causing mutation in exon 42 that was noted above, and the other was a donor splice site mutation in intron 10 (c.1356+1G > A). Patient STG-1 harbored a splice site mutation (c.5836-2A > G) in intron 41 and a variation in exon 6 that causes p.Arg212His [Table 1]. The intronic mutation is a known STGD1-causing mutation. As the p.Arg212His-causing variation was observed in the homozygous state, the allele with the c.5836-2A > G intronic mutation must be in cis with a c.635G > A variation that causes p.Arg212His. Although the p.Arg212His-causing variation has sometimes been considered a polymorphism because of its relatively high allele frequency (0.052720), it was reported to contribute to STGD1 status in a Turkish patient.^[4,7,18,19]^ It is possible that in an individual with a clearly disease-causing mutation such as the splice site mutation of patient STG-1, the presence of p.Arg212His will result in disease presentation. Alternatively, the second mutated *ABCA4* allele in patient STG-1 may be positioned in one of the many exons not screened in this study. Patient STG-5 also harbored two variations in *ABCD4*, a variation that causes p.Gly1961Glu and the intronic variation c.1356+11T > G. Although the intronic mutation is rare (0.0001), bioinformatics tools including Human Splice Finder (http://umd.be/Redirect.html) and NNsplice (https://www.fruitfly.org/seq_tools/splice.html) predict that it would not affect splicing. Therefore, the second mutated *ABCA4* allele in patient STG-5 is likely positioned in one of the exons not screened. The presence of a shared haplotype between the two mutated alleles that cause p.Gly1961Glu cannot be ascertained because of phase issues in the two heterozygous carriers, STG-5 and STG-6. Sequence chromatographs of the reported STGD1-associated mutations are shown in Figure 2.

##  DISCUSSION

All diseases associated with *ABCA4* are progressive retinopathies accompanied by degeneration of photoreceptor cells that can lead to blindness. There is a significant association between the severity of phenotype and the nature of the *ABCA4* mutations. Stargardt disease is generally considered to be less severe than cone-rod dystrophy (CRD) or retinitis pigmentosa (RP).^[20,21]^ Deletions, nonsense mutations, and INDELs are usually associated with severe phenotypes.^[5]^ Missense mutations are more commonly found in less severely affected patients. It is interesting that the pathogenicity of some missense mutations, such as p.His432Arg and p.Arg212His discussed above, and even of p.Gly1961Glu that is described below, remains controversial.

**Table 1:** Data on patients with at least one candidate Stargardt disease-causing mutation in ABCA4


**Patient ID**	**Sex**	**Consan- guinoeus parents**	**AOS**	**AAE**	**Visual acuity**		**Candidate disease-causing variations**							**Non-disease associated variations**				

			**OD**	**OS**	**Variation**	**Homo/ Het**	**Exon/ Intron**	**Effect**	**MAF gnomAD**	**ACMG annnotation**	**Variation ID**	**Variation**	**Homo/ Het**	**MAF gnomAD**	**ACMG annnotation**	**Variation ID**
STG-2	F	+ 6	8	20/200	20/200	c.5881G>A	Homo	Exon 42	p.Gly1961Arg	0.0001	Likely pathogenic	rs142253670			
STG-6	F	–	29	31	1/10	1/10	c. 1356+1G>A	Het	Intron 10	Splicing	No data	Novel	c.302+26A> G	Het	0.49537	No data	rs2297634
				c.5882G>A	Het	Exon42	p.Gly1961Glu	0.0035	Likely pathogenic	rs1800553	c.5836 -11G>A	Het	0.2035	No data	rs1800739
							c.5844A>G	Homo	0.1978	Benign	rs2275029
STG-18	M	+ 6	37	20/150	20/200	c.1648G>A	Homo	Exon 12	p.Gly550Arg	0.000004	Uncertain significance	rs61748558			
STG-1	F	–	12	20	20/160	20/160	c.635G>A*	Homo	Exon 6	p.Arg212His*	0.05272	Benign	rs6657239			
				c.5836-2A> G	Het	Intron 41	Splicing	No data	CS161784			
STG-5	F	–	16	17	CF at 500 cm	CF at 400 cm	c.5882G>A	Het	Exon42	p.Gly1961Glu	0.0035	Likely pathogenic	rs1800553	c.1356+11T>G	Het	0.000137	No data	rs113055350

*Considered to possibly contribute to disease status as describred in the text F, female; M, male; AOS, age at onset; AAE, age at examination; OD, right eye; OS, left eye; CF, counting finger; Homo, homozygous; Het, heterozygous; MAF, minor allele frequency																		

One or more *ABCA4*-mutated alleles were identified in 5 of the 18 Iranian STGD1 patients (27.8%) in whom only six of the gene's 50 exons were screened. No variant nucleotide was found in exons 3 and 13, and the single variation found by screening exon 10 was in fact in intron 10. Without considering p.Arg212His as a mutation that affects disease status, five different mutations were observed. Three of the mutations were missense mutations. P.Gly1961Glu and p.Gly1961Arg affect an amino acid that is localized in the second NBD domain. Although p.Gly1961Glu has been reported as one of the most frequent mutations in Stargardt patients of various populations, its pathogenicity has been questioned largely because of the relatively high frequency of its coding allele in some populations.^[22]^ For example, its frequencies in the Somalian, Ashkenazi, Qatar, and Iranian populations are reported to be 0.10,^[22]^ 0.024 (gnomAD), 0.023,^[23]^ and 0.026 (Iranome), respectively. The consensus appears to be that p.Gly1961Glu is a moderate mutation.^[5,24]^ In the homozygous state, it presents a mild form of STGD1; in the compound heterozygous state with a more deleterious mutation, it can contribute to a severe form of Stargardt disease.^[5]^ As in other populations, p.Gly1961Glu may be relatively common among Iranian STGD1 patients, as it was observed in two patients of the relatively small cohort studied here. P.Gly1961Arg is found less frequently than p.Gly1961Glu in retinal dystrophy patients. The frequency of the allele that causes p.Gly1961Arg among Iranians is 0.0025, and the frequency is < 0.01 in most other populations as well (Iranome, gnomeAD). P.Gly550Arg, that has been reported as a Stargardt disease-causative mutation, was the third missense mutation found in the Iranian cohort.^[25]^ P.Gly550 is localized in the extracytosolic domain 1 (ECD1) of the protein encoded by *ABCA4*.

The molecular consequences of two observed splice site mutations were not critically investigated. C.5836-2A > G that abolishes the acceptor splice site in intron 41 was previously reported in a CRD patient of a Chinese cohort.^[6]^ C.1356+1G > A is being reported for the first time in a Stargardt disease-affected patient, although c.1356+1G > T was previously reported in age-related macular degeneration patients.^[26]^ This signifies potential variability of phenotypic features associated with any *ABCA4* mutation.

To the best of our knowledge, this is the first report of the *ABCA4 *mutation screening of the Iranian patients affected by Stargardt disease. Of course, more patients need to be screened in order to achieve a representative profile of *ABCA4 *mutation in this population. This knowledge will be needed for various purposes, including possible initiatives for gene therapy.

##  Financial Support and Sponsorship

Nil.

##  Conflicts of Interest

There are no conflicts of interest.
